# Tracking the evolution of medical students' clinical documentation skills: a pilot study leveraging a simulated electronic record and a longitudinal panel data analysis

**DOI:** 10.3389/fmed.2026.1829687

**Published:** 2026-05-29

**Authors:** Zakia Dimassi, Mohammed Abuzitoon, Albert Wijeweera, Chandana Wijeweera, Masood Ahmad, David Murray, Salman Yousuf Guraya

**Affiliations:** 1 Department of Medicine and Health Sciences, Khalifa University, Abu Dhabi, United Arab Emirates; 2 College of Medicine and Health Sciences, United Arab Emirates University, Al Ain, Abu Dhabi, United Arab Emirates; 3 Department of Management, Science and Engineering, Khalifa University, Abu Dhabi, United Arab Emirates; 4 Department of Medicine, Nursing and Health Sciences, Monash University, Melbourne, VIC, Australia; 5 Sheikh Shakhbout Medical City (SSMC), Abu Dhabi, United Arab Emirates; 6 Department of Medicine, University of Iowa, Iowa City, IA, United States; 7 Khalifa University Abu Dhabi, Abu Dhabi, United Arab Emirates; 8 College of Medicine, Gulf Medical University, Ajman, United Arab Emirates

**Keywords:** clinical documentation skills, clinical, documentation, entrustable professional activities, EPAS, medical education, panel data analysis, simulation

## Abstract

**Background:**

Clinical documentation skills (CDS) are crucial for effective medical practice. Accurate and structured documentation ensures continuity of care, supports clinical decision-making, and serves as a legal record of healthcare delivery. This study aimed to evaluate the progression of CDS of the medical students across core clinical rotations using a simulation-based modified Standardized Long Case Examination of Clinical Competence (m-SLCECC).

**Methods:**

We performed a longitudinal analysis of the progression of 3^rd^ year medical students' CDS on the history of present illness (HPI), and assessment and plan (A&P) using simulation-based assessment in five clinical disciplines over seven months. During the simulation-based clinical assessment of m-SLCECC, students completed post-encounter documentation tasks. Data were collected via the LearningSpace™ system and analyzed using pooled panel data estimation. Three separate regression models evaluated improvements in HPI, plan, and assessment documentation scores over time, adjusting for clerkship- and case-specific variables.

**Results:**

The medical students' CDS scores improved across all domains over the study period. HPI scores increased by 0.17 points per month (*p* = 0.01), assessment by 0.15 points (*p* = 0.02), and planning scores by 0.22 points (*p* < 0.001). Assessment scores showed the greatest variability (R^2^ = 0.42) compared to HPI (R^2^ = 0.20) and plan (R^2^ = 0.27). However, clerkship-specific variations and differences in case content influenced the degree of improvement.

**Conclusion:**

Simulation-based longitudinal assessment effectively captured progressive improvements in students' CDS. Variations in performance highlight the influence of rotation-specific factors and underscore the need for targeted feedback strategies to optimize CDS development. Panel data analysis in this study proved a robust methodological tool for tracking skill evolution over time.

## 1 Introduction

Clinical documentation skills (CDS) constitute a foundational component of effective medical practice. Accurate, structured, and comprehensive documentation not only supports clinical decision-making but also ensures continuity of care, enhances interprofessional communication, and fulfills essential medico-legal responsibilities (1, 2). In the existing literature on the subject, some studies have highlighted the importance of adequate clinical documentation (3, 4) while others have critiqued the time spent on the documentation of clinical encounters (5, 6). Recently, we have witnessed the adoption of sophisticated statistical methods to assess the training outcomes of medical students for the use of electronic health records (EHR), while maintaining patient interactions (7); whereas some researchers have compared medical students' clinical skills performance across different skills assessed in objective structured clinical skills (OSCE) (8). Interestingly, the study by Violato et al. used an analysis of multivariate hierarchical linear modeling to determine the fit of learning curve models of negative exponential growth to repeated measures of performance based on entrustable professional activities (EPAs) (9).

Despite the centrality of this competency, CDS have historically received less curricular emphasis compared to diagnostic reasoning or procedural training, resulting in medical graduates lacking proficiency in structured notetaking, synthesizing key clinical information, and maintaining consistency within electronic health records (EHRs) (10, 11). These deficiencies raise concerns regarding graduates' readiness for independent practice, particularly given the well-documented links between poor documentation, medical errors, miscommunication, and inefficiencies in healthcare delivery (3–6).

Recognizing these challenges, the Alliance for Clinical Education has reaffirmed the importance of clinical documentation as a core competency that requires high-quality supervision and feedback (4, 12). Nevertheless, the implementation of CDS training remains fragmented and hindered by limited integration across educational infrastructure and clinical rotations (5, 6). Structured, scalable, and evidence-based educational approaches are therefore necessary to establish strong foundation for longitudinal teaching and assessment of documentation competency (3, 4, 13).

Our original study aimed at examining the CDS of the medical students at Khalifa University—College of Medicine and Health Sciences (KUCMHS)—United Arab Emirates (UAE), benchmarked against the relevant EPA framework by the Association of American Medical Colleges (AAMC) (14). Using the pooled panel data estimation method, we tracked the development of clinical medical students' post-encounter documentation of three competencies of history of present illness (HPI), assessment, and planning on the modified standardized long-case examination of clinical competence (m-SLCECC) (15, 16). Incorporating the simulation-based standardized clinical assessments offer a structured and repeatable mechanism to track CDS progression over (17). The findings of the study showcase valuable data that can encourage medical educators to integrate formal assessment of CDS of medical students and to track their progress over time. By illustrating how pooled panel methods can be leveraged with simulation-based assessment data, this study offers a framework for monitoring CDS development and informing the design of longitudinal documentation training within simulation-enhanced undergraduate medical curricula.

## 2 Methods

This study was conducted at KUCMHS in the UAE on a target population of 27 third-year medical students enrolled in the Doctor of Medicine program for the academic year 2021–2022. The clinical skills curriculum of this program was built around the 13 core EPAs (14). The performance of these students on simulation-based modified Standardized Long Case Examination of Clinical Competence (m-SLCECC) assessments was followed between June 2021–December 2021 as they undertook their core clinical rotations in Family Medicine (FM), Internal Medicine (IM), Obstetrics and Gynecology (OBGYN), Neurology (NEU), Pediatrics (PED), and Surgery. This design allows each simulated encounter to function as a repeated, criterion-based opportunity to demonstrate CDS within a controlled, reproducible environment (3, 6). Psychiatry exams were not included as they follow a different assessment format than the rest of the clinical rotations. As a part of their clinical skills training, these students were taught the basics of post-clinical encounter documentation following the subjective, objective, assessment, plan (SOAP) note model, and the onset, location, duration, characteristics, aggravating/relieving, timing, and severity (OLDCARTS) mnemonic for exploring the HPI.

As our objective was to conduct a pilot project aimed at characterizing the development of CDS within the KUCMHS MD curriculum, we limited the target population to the 3^rd^ year MD medical students undertaking core clinical rotations. During these clinical placements, each student completes up to 14 formative and summative m-SLCECC examinations per year (including Psychiatry). Given the relatively small size of the eligible cohort (27 students during the study period) and our primary interest of an in-depth, longitudinal analysis of all available m-SLCECC encounters rather than in estimating parameters for a broader population, we used purposive sampling to recruit participants (18). This sampling strategy, commonly employed in single-institution medical education research when the unit of interest is a specific cohort, enabled us to obtain a substantive volume of repeated assessments per learner.

All 27 third-year medical students who were actively enrolled in the KUCMHS MD program for the academic year 2021–2022 provided written informed consent (S2 (1) Written Informed Consent) to participate in the study. The study was approved and deemed exempt by the Research Ethics Committee of Khalifa University of Science and Technology under Protocol H24-012 (S2 (2) KU-IRB approval).

To assess medical students' key clinical skills, we developed a modified version of the Standardized Long Case Examination of Clinical Competence (m-SLCECC) (15) targeting EPA 1 (gather a history and perform a physical examination), EPA 2 (prioritize a differential diagnosis following a clinical encounter), EPA 3 (recommend and interpret common diagnostic and screening tests), and EPA 5 (document a clinical encounter in the patient records) (14). The latter was specifically included based on input from clinical clerkship directors on the medical students' inadequate skills in recording patient notes, as well as the program's focus on readying international medical graduates (IMGs) for their transition into residency training in the USA.

### 2.1 Exam logistics

The m-SLCECC is scheduled per clinical clerkship, and we planned multiple assessments (both formative and summative) of different clinical clerkships on the same day. One week prior to the examination, the staff's proctoring assignments were circulated (Table 1).

**Table 1:** Staff role assignment for assessment of clinical competencies.

Formative or summative clerkship assessment		
Role	Time	Person
Exam lead	7:45	TG
Faculty coordination	7:45	TG
Student attendance/check in and out	7:45	OME
SP trainer	7:45	SK/TG/SA
SP coordinator	7:45	SK/TG/SA
Door note–all clerkship	7:45	TV
Simulator orientation–peds HAL	7:45	AM

All students received detailed instructions on the schedule of the clinical assessment, as detailed in Table 2.

**Table 2:** Schedule and debriefing plan for clinical assessment.

Date/Formative or Summative/Clerkships					
STATIONS			DOCUMENTATION		
	Exam room number	Exam room number		Room 10 (External & Internal)	Room 9 Internal & Room 8 External
Case	Case title	Case title	Post Encounter documentation for 20 min at the computer station.		
Assessor	Clinical Faculty Name	Clinical Faculty Name			
SP	SP Name	SP Name			
**8:00 AM**	**Reporting Time - Simulator Orientation in Room 7 for all students**				
8:43 AM	**For All Students - Kindly arrive at your respective Exam Stations, only 2 min before your allocated time to read the door note**				
8:45 AM	Student 1	Student 3	9:07–9:27	Student 1	Student 3
9:07 AM	Student 2	Student 4	9:29–9:49	Student 2	Student 4
9:29 AM	Student 3	Student 1	9:51–10:11	Student 1	Student 3
9:51 AM	Student 4	Student 2	10:13–10:33	Student 2	Student 4
10:13 AM	Student 5	Student 7	10:35–10:55	Student 5	Student 7
10:35 AM	Student 6	Student 8	10:57–11:17	Student 6	Student 8
10:57 AM	Student 7	Student 5	11:19–11::39	Student 5	Student 7
11:19 AM	Student 8	Student 6	11:41–12:01	Student 6	Student 8
11:39 AM	End of Exam		12:01	End of Documentation	
(^*^Kindly discuss with your clerkship director)–FEEDBACK BEGINS 12:10 pm - DEBRIEFING ROOM 4					
Announcements					
0:00	Learners, you may begin				
0:13	2 min remaining				
0:15	Learners, you may exit the room				

Student Briefing: This is a 2 station Exam. Each station lasts 15 min. You will receive a 2-min warning at 13 min. Please make sure you can log in to the Learning Space System before the Exam. Learning Space System–https://learningspace.ku.ac.ae/ READ THE DOOR NOTE CAREFULLY. It will explain the tasks you are required to perform. Your assessor will not be in the room. Ensure that your voice is projected to be heard. “This is a closed-book test, and you are to complete it by yourself without consulting any resources. Please refer to academic integrity policy for consequences of academic dishonesty.”

### 2.2 Development of assessment plan

For each rotation, a pool of m-SLCECC stations was developed, following proper exam content mapping to the curriculum and direct involvement of subject matter experts (SMEs) in assessment, both of which are fundamental to content validity. A combination of two stations was selected for each exam to reflect the targeted core competencies (history-taking skills, verbal and nonverbal communication skills, physical examination skills, and CDS) and the objectives of the clerkship. Each station comprised history +/- physical examination skills (EPA 1), and CDS (EPA 5). The simulation scenarios were co-developed by the clinical clerkship directors and the clinical skills team (CST). Subsequently, the standardized patients (SPs) and the simulation equipment with the appropriate profiles were identified. Students' performance was assessed using a combination of item-based rating rubrics, completed by the SME, and global rating scales of medical students' communication skills, using a modified version of the Master Interview Rating Scale (MIRS) (originally developed by Pfeiffer C.A.) and completed by the SPs. Both rubrics were incorporated into LearningSpace™ (LS).

### 2.3 Simulation integration

A diverse range of simulation modalities, including structured debriefing sessions, were integrated into all the m-SCLECC to enhance the realism and immersive characteristics of the experience. In the PED clerkship, simulation modalities included SPs as human simulators, low-fidelity full-body mannequins, and hybrid simulations combining SPs with high-fidelity mannequins. In FM and IM clerkships, a station was created by combining a chest auscultation part-task trainer with both an SP and a screen-based simulation, in which students interpreted vital signs displayed on a simulated patient monitor. In the SUR clerkship, moulages were used to replicate post-operative surgical sites. In the OBGYN clerkship, students were engaged with multiple modalities within a single station, including part-task trainers (PTT), SPs, and hybrid simulation, for comprehensive learning experience. In the NEU clerkship, SPs served as the primary simulation modality, allowing students to refine their neurological examination and diagnostic reasoning skills through direct patient interaction.

### 2.4 Assessment workflow

**Figure 1:**
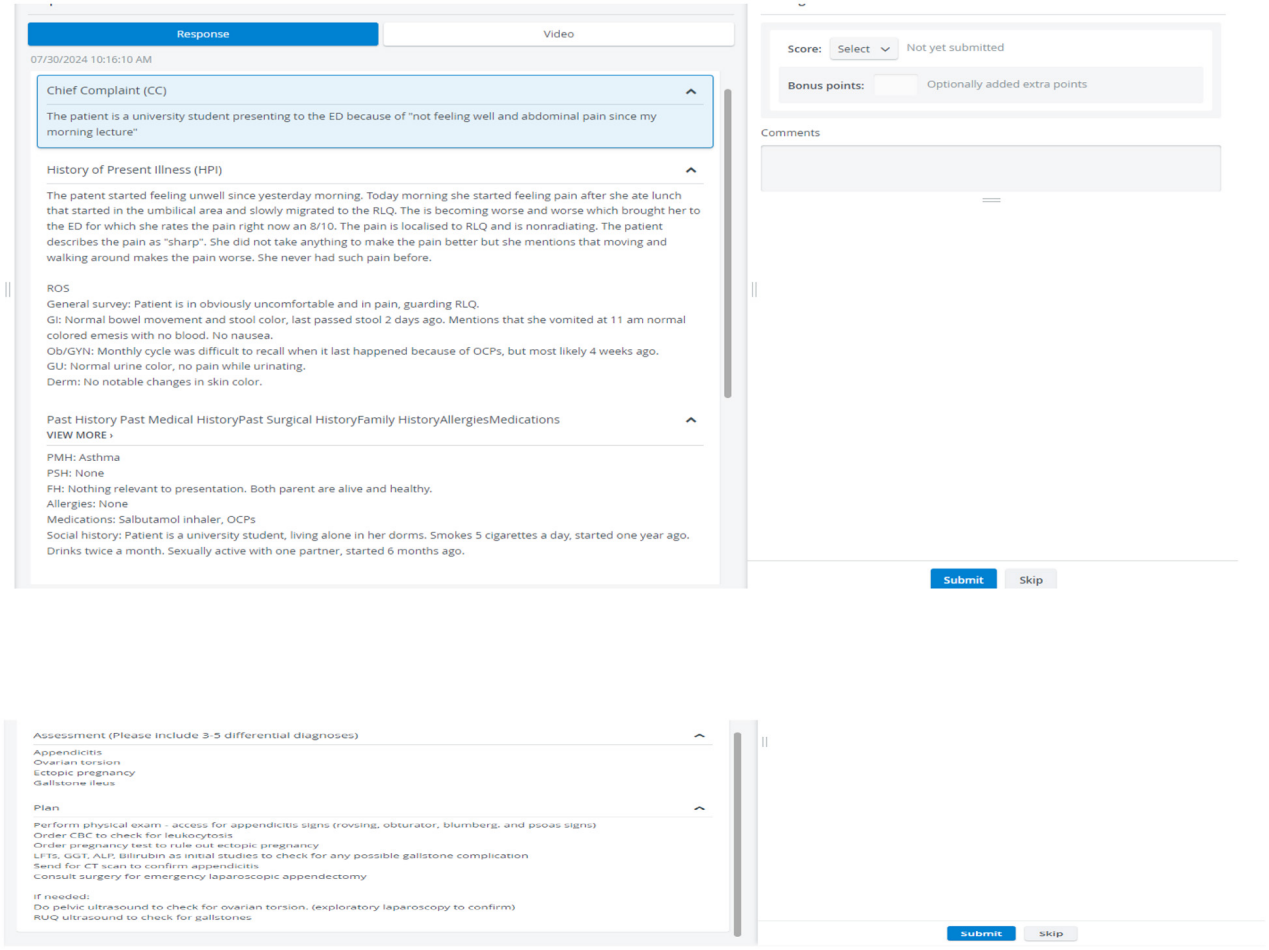
A screenshot from a student's LearningSpace™ post-clinical encounter note.

The m-SCLECC followed a structured, multi-station designe to assess students' clinical competencies in a standardized environment. Before entering a given station, the students were gathered in a designated corridor, equipped with PC all-in-one screens displaying the door note, which outlined the clinical scenario, required tasks, and station duration. The Learning Management System (LMS) LS™ provided automated voice announcements to guide students through the process, including entry instructions, time-remaining reminders (e.g., “five min remaining”), and prompts to exit or transition to the next station. Each assessment comprised two stations, and each station lasted between 40 and 50 min, with the first 15–30 min spent inside the exam room where the students took history and/or performed physical examination on either the SP or PTT. The students would then exit the room and return to the corridor, where they were tasked with documenting clinical encounters for 20 min, with an additional 50% time allowed for students with special educational needs. Students logged into their personal LS™ accounts and accessed the data entry page, which was divided into sections following the SOAP note structure (Figure 1). After performing the post-encounter clinical documentation, students moved to the following station and repeated the process until all stations were completed.

### 2.5 Assessment of clinical performance

The assessment of clinical performance was based on the direct (real-time or recorded) observation of the students' performance on history-taking (20%), physical exam skills (20%), and documentation skills (40%) by an SME, and assessment of communication skills by the SP (20%). The SP encounter rating rubric was developed based on the Kalamazoo Consensus Statement (19) as well as content-specific elements of history and physical exam. It comprises numerical scores assigned to each item, similar to the scoring rubric described by Harden (14). The number of items varied depending on the station's objectives and complexity and was limited to a maximum of 25. The response scale for each item was built with a binary option: (1) performed: score 1, and (2) not performed: score 0. The SP rating scale was based on a modified version of the MIRS. The documentation rating rubric was constructed using a combination of the Patient-Centered Documentation Assessment Rubric for the HPI portion of the SOAP note (20), and a standardized rubric based on the five key components of SOAP writing (subjective and objective findings, physical exam, assessment [problems and differentials], and plan); the purpose of which is to quantify students' clinical and diagnostic reasoning as depicted in their post-encounter notes (21). The assessor was instructed to assign a numerical score based on the completeness of the post-encounter note relative to the clinical scenario and to provide a narrative assessment of the students' performance.

### 2.6 Data collection and evaluation process

#### 2.6.1 Data collection

We examined students' CDS on the m-SLCECC during the core third-year clinical rotations, focusing on the HPI, assessment, and plan sections, aiming to capture a substantial aspect of the students' clinical reasoning and decision-making abilities (22). Assessment data from the post-encounter notes were collected during the summative m-SLCECC at the conclusion of each clinical rotation for all 27 students, over a seven-month period (June to December), capturing multiple assessments per student over time.

#### 2.6.2 Randomization of raters

Using Microsoft Excel's built-in RAND and RANDBETWEEN functions, each student's specific documentation was assigned a unique identifier by one of the co-authors (MAZ). This randomization was applied both to the assignment of students' assessments to the rater, and the specific documents to minimize bias (23), which should enhance the study's validity and generalizability (24).

#### 2.6.3 Standard setting and calibration

Adapted to the grading approach described by Gleeson (25) the assessment grading was conducted by three independent raters (DM, MA, and ZD), where each assessor was randomly assigned one component of a student's documentation. Rater one focused on assessing the HPI while raters two and three evaluated the assessment and plan. The raters were blinded to the students' identities and to each other's assessments, ensuring that each documentation component was scored solely on its own merits. Prior to the scoring process, the three raters conducted standard setting and a pilot video-based calibration on how to grade each competency, ranging from 0 to 10, then independently rated the same set of pilot documentation cases in a blinded protocol. These scores were compared and discussed in a structured calibration session led by the lead examiner (DM), where differences in interpretation were explored, and consensus was reached on scoring standards. Additional cases were reviewed during the calibration process to reinforce consistency in scoring. Once agreement was established, scoring responsibilities were divided by domain and clerkship to maintain efficiency. This process helped faculty identify nuances in clinical performance and standardize scoring. Group discussions resolved discrepancies, clarified judgments, and built consensus, improving inter-rater reliability and highlighting possible rater bias or error.

#### 2.6.4 Statistical analysis

Given the structure of the dataset and the objectives of the study, a pooled regression framework was considered the most appropriate analytical approach. This method offers several key advantages, including studying the dynamics of change with short time series, combining time series with cross-sections and therefore enhancing the quality and quantity of the data compared to only using one of these two dimensions, either cross section or the time dimension (25, 26).

Although fixed- and random-effects models are commonly used for panel data, their application in this context is constrained by the relatively small number of cross-sectional units. Estimating individual-specific effects under such conditions may lead to inefficient or unstable estimates and potential overparameterization.

As this study aimed to evaluate medical students' clinical skills across disciplines and over time, we estimated three distinct models, each corresponding to a different performance metric in post-encounter documentation: Model one assessed the grades of HPI, model two assessed the grades of the assessment, and model three evaluated the grades of the plan.

Each model includes a set of independent variables drawn from three main categories: seven core clinical rotation subjects, two case-related variables, and a time variable. As noted earlier, the seven core clinical clerkships are Family Medicine (FM), Internal Medicine (IM), Neurology (NEU), Obstetrics and Gynecology (OBG), Pediatrics (PED), and Surgery. However, all three models include only six clinical rotation variables, as Surgery is used as the reference category (27). Similarly, to account for the two case-related variables (Case 1 and Case 2), only one explanatory variable, clinical documentation skills (CDS), is included in each model, with Case 2 serving as the reference category. Because assessments were conducted over a 7-month period, the time variable MONTH is included to capture temporal effects on performance.

## 3 Results

We analyzed post-encounter documentation notes from the summative m-SLCECC examinations administered at the end of each clinical rotation for all 27 students with 255 data points, (9 males and 18 females; F:M = 2:1), with a mean age of approximately 25 years. However, our panel is unbalanced, with only 255 observations, as the number of assessments taken by each student varied throughout the study period.

The estimated results from the three pooled panel performance models are reported in Table 3.

**Table 3:** Pooled estimation results of the grades of medical students to determine the progress of their post-encounter clinical documentation skills.

Variable	Model 1-HPI			Model 2-assessment			Model 3-plan		
	Coefficient	*t*-stat	*P*-value	Coefficient	*t*-stat	*P*-value	Coefficient	*t*-stat	*P*-value
**C**	4.83	14.04	0.00^*^	4.24	11.96	0.00^*^	4.18	13.29	0.00^*^
**CSD**	-0.03	−0.14	0.89	0.38	1.66	0.10	0.25	1.08	0.28
**FM**	0.89	2.57	0.01^*^	−0.73	-1.85	0.06	-0.77	−2.02	0.04^*^
**IM**	-1.32	−3.70	0.00^*^	1.81	5.73	0.00^*^	1.58	5.16	0.00^*^
**NEU**	-0.98	−2.32	0.02^*^	2.15	6.19	0.00^*^	1.41	3.14	0.00^*^
**OBG**	-0.54	−1.58	0.12	−1.79	-4.89	0.00^*^	-0.74	−2.14	0.03^*^
**PED**	0.46	1.24	0.22	−0.34	-0.89	0.37	-0.57	−1.70	0.09
**MONTH**	0.17	2.48	0.01^*^	0.15	2.34	0.02^*^	0.22	3.19	0.00^*^
	R-squared = 0.20			R-squared = 0.42			R-squared = 0.27		
	F-Statistic = 8.77			F-Statistic = 25.27			F-Statistic = 12.92		

HPI, history of present illness; CSD, clinical documentation skills; FM, Family Medicine; IM, Internal Medicine; NEU, Neurology; OBG, Ob-Gyn; PED, Pediatrics; MONTH, month of the academic year during the study period.

^*^p-value that is statistically significant (< 0.05).

Across all three models, the coefficient on the MONTH variable was positive and statistically significant, indicating a consistent improvement in students' performance over time. For example, in Model 1, holding other factors constant, the HPI score increased by an average of 0.17 points with each subsequent assessment. Similarly, Models 2 and 3 showed incremental gains of 0.15 points in assessment scores and 0.22 points in plan scores, respectively. These findings suggest a general longitudinal improvement in clinical documentation skills as students progressed through successive rotations, rather than improvements attributable to any single instructional context.

The estimated effects of the case variable (CSD) exhibited a mixed pattern across models. Model 1 yielded a negative coefficient, whereas Models 2 and 3 showed positive coefficients when students transitioned from case one to case two. However, statistical significance was observed only in Model 2. Taken together, these results provide limited evidence that performance may vary across cases, potentially reflecting differences in case complexity, familiarity with assessment formats, or timing within the academic year, rather than systematic differences in student competence.

The coefficients associated with specific clinical clerkships also varied across models and performance domains. While some clerkship indicators were associated with positive coefficients and others with negative coefficients, these estimates should not be interpreted as reflecting inherent strengths or weaknesses of particular rotations. Instead, they likely capture a combination of unobserved factors, including variation in case mix, assessment timing, instructional emphasis, and clinical workload across clerkships. For instance, the negative coefficient observed for Internal Medicine in Model 1 (a 1.32-point lower average HPI score relative to the reference category) and the positive coefficient for Family Medicine (0.89-point increase) should be understood as relative associations within the pooled model, rather than as evaluative judgments about clerkship quality or effectiveness. Similar variability across clerkships was observed in Models 2 and 3, underscoring the contextual and multifactorial nature of clinical skills development.

The adjusted R-squared values are reported in the lower panel of Table 3. The three pooled regression models demonstrate varying levels of explanatory power. Model 1, which examines student performance in HPI, has an adjusted R-squared of 0.20, indicating modest explanatory ability. Model 2, focused on performance in assessment, shows the highest explanatory power, with an adjusted R-squared of 0.42, suggesting that the included variables account for a substantial proportion of the variation in outcomes. Model 3, which analyses performance in the plan component, has an adjusted R-squared of 0.27, reflecting a moderate level of explanatory strength. Overall, the models indicate that the predictors are most effective in explaining variation in assessment performance compared to the HPI and plan components.

**Figure 2:**
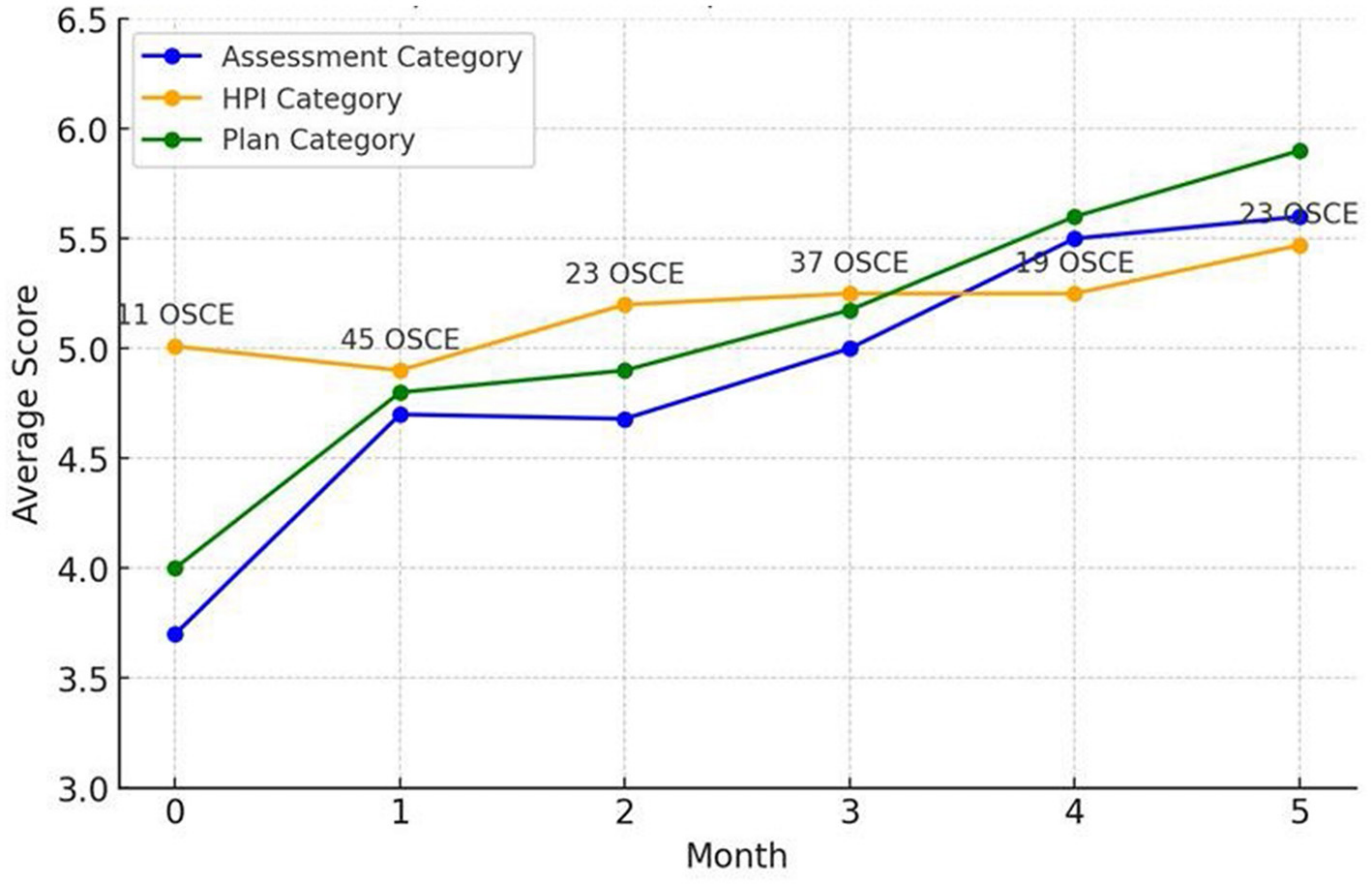
Predicted mean performance scores in various clinical documentation competencies over time.

**Figure 3:**
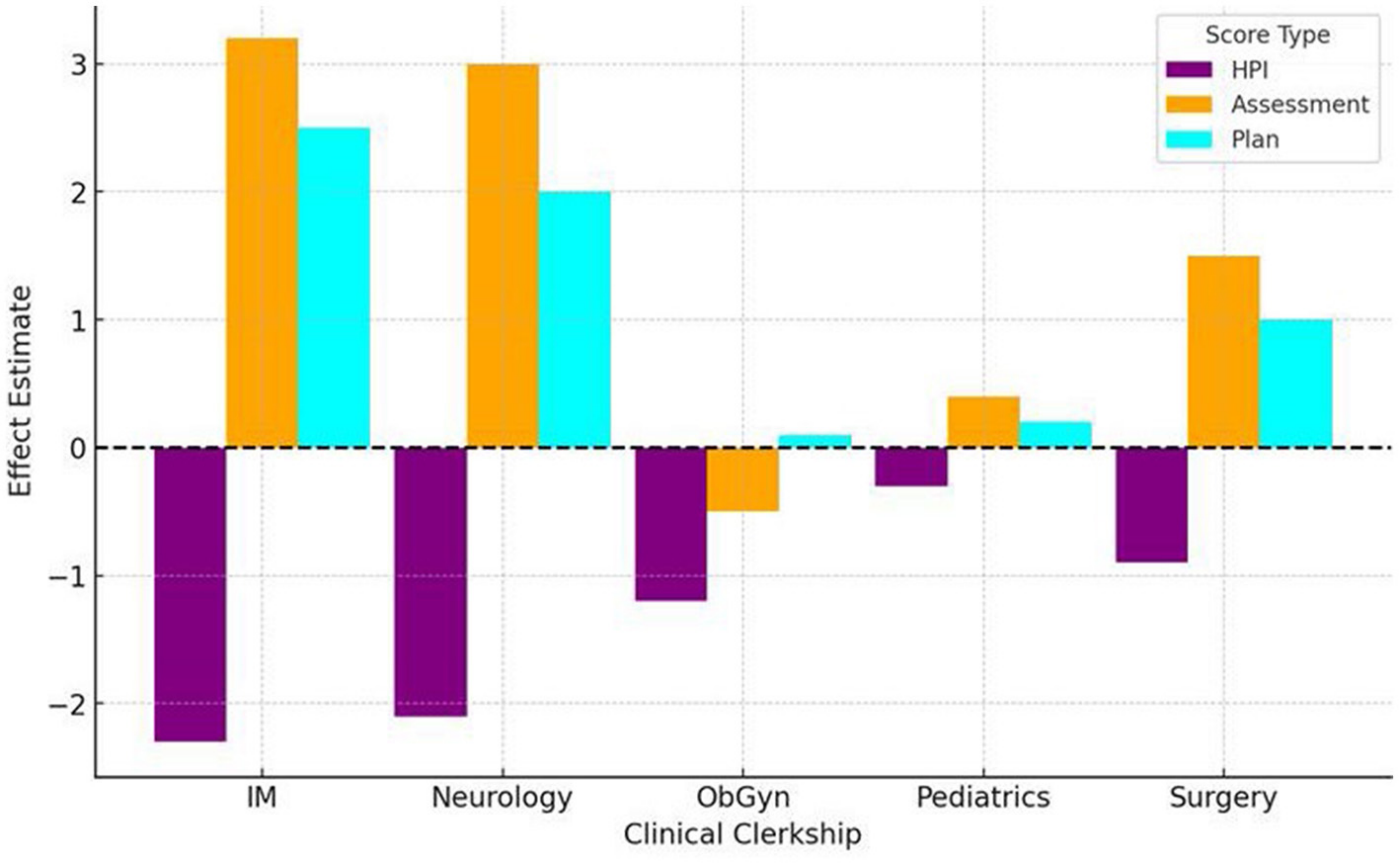
Effect of clinical clerkships on Clinical Documentation Skills (CDS) on history of present illness (HPI), assessment, and plan scores across the five core Medicine3 clerkships.

To complement the regression results, we predicted mean performance scores across the three competencies HPI, assessment, and plan illustrated in Figure 2. These predicted values reveal a clear upward trajectory in the mean student performance over time, with the largest proportional improvement observed in the plan domain (33%), followed by assessment (24.3%) and HPI (20.4%). Figure 3 presents the estimated clerkship-specific effects relative to Surgery (the reference category), highlighting variability in estimated associations across ObGyn, Pediatrics, and Surgery. Consistent with the regression analysis, these differences are interpreted as reflecting contextual and curricular variation across clerkships rather than intrinsic differences in educational quality.

## 4 Discussion

This pilot study used a longitudinal, simulation-based assessment embedded within a modified Standardized Long Case Examination of Clinical Competence (m-SLCECC) to track the evolution of third-year medical students' CDS across five core clerkships. Our study examined overall trends in students' performance across repeated observations rather than isolating unobserved individual heterogeneity. As such, incorporating fixed or random effects was not central to the research question. Using a pooled panel-data approach, we observed progressive improvement over seven months across all three targeted domains: HPI, assessment, and plan, with the highest gains in plan, followed by assessment, and more modest yet significant gains in HPI. In our study, the pooled estimation approach was particularly appropriate due to the relatively small number of students. By observing students' performance over multiple periods, the analysis expands the dataset to 255 data points. This approach increased the estimation efficiency by reducing standard errors through a larger number of observations and mitigated the omitted variable bias by allowing the inclusion of more relevant independent variables. Our findings are consistent with the general understanding that repeated exposure to clinical encounters and structured assessment opportunities support incremental clinical skill acquisition over time, even in a relatively small cohort.

The differing trajectories across domains provided insights into how CDS develop within a simulation-enhanced curriculum. The more rapid growth in assessment and plan scores relative to HPI suggested that students may have been increasingly able to translate clinical data into diagnostic impressions and management strategies, even as foundational information-gathering and narrative synthesis skills improved at a slower pace. This pattern aligned with prior work on clinical milestones and learning curves, which described early variability and nonlinear growth before performance plateaus (28, 29). In our context, the relatively slower progress in HPI documentation might reflect both the complexity of constructing a coherent, patient-centered narrative and the lingering impact of pandemic-related reductions in authentic clinical exposure for the study cohort. In addition, the absence of consistent, structured feedback specifically targeting the reasoning embedded in students' written notes might have contributed to the observed mismatch between their performance during clinical encounters and the depth of explanation captured in their documentation (10).

Clerkship-specific differences further underscored the role of content and context specificity in CDS development. The pooled models and predicted trajectories indicated that some rotations were associated with higher average documentation scores than others, even after adjusting for time and case characteristics. These patterns were consistent with theories of situated learning and context specificity, which emphasized that performance reflects an interaction between learner characteristics, case features, supervision, and assessment design rather than a purely trait like “ability” (28–31). Lower HPI scores observed in Internal Medicine, for example, might signal differences in case complexity, expectations for diagnostic reasoning, or the quality and timing of feedback rather than inherent student deficits. For simulation educators, these findings highlight the importance of aligning station design, debriefing structures, and feedback practices across clerkships to promote more consistent opportunities to practice and refine CDS.

Conceptually, the documentation rubric used in this study operationalized EPA 5 in ways that closely overlap with clinical reasoning: filtering and organizing information, constructing a coherent narrative, prioritizing problems, articulating working and differential diagnoses, justifying decisions, and documenting patient preferences (10, 32). The observation that post-encounter notes often did not fully reflect the level of competence inferred from live performance reinforced the importance of “making thinking visible” in both simulation and workplace-based assessment (WBA). Regarding clinical documentation, specifically the assessment & plan section, debriefing and feedback should not focus solely on observable behaviors in the encounter but also on how learners provide explicit explanations and justifications for the order of the differential diagnosis and the choices of interventions. Structured tools, such as post encounter forms or guided self-explanation tasks, may help bridge this gap (28, 30, 33).

Finally, this work has implications beyond a single institution. The post encounter patient note was a central feature of the former United States Medical Licensure Exam (USMLE) Step 2 Clinical Skills examination, and its discontinuation has created an assessment gap, particularly for international medical graduates (IMGs) seeking to demonstrate readiness to practice in new health systems (13, 34). Our findings suggest that simulation-based, EHR-embedded documentation exercises aligned with EPA5 can serve as one component of evidence-informed assessment strategy for CDS. When integrated with WBA and other forms of programmatic assessment, longitudinal simulation data of the type generated by the m-SLCECC may help schools monitor students' preparedness for residency-level documentation demands and support targeted remediation, including for IMGs who may face additional acculturation challenges in documentation practices (31).

Overall, this pilot study showed that a simulation-based, longitudinal assessment approach can both support learning and generate high yield data about how CDS develop over time. For those designing and studying simulation curricula, the combination of whole task standardized encounters, simulated EHR documentation, and panel data analysis offers a promising approach to understanding and optimizing documentation training within competency based medical education.

### 4.1 Limitations

As this study was based on a pilot project, it inevitably had several limitations that should be considered when interpreting the findings. First, our project was limited to a small number of students in a single institution. This limitation was compensated for by using repeated measures that expanded the dataset to 255 observations, improved statistical efficiency, and produced statistically significant findings. Nevertheless, these findings should be interpreted as exploratory.

Second, our measures were restricted to performance on summative m-SLCECC and did not include other components of students' assessment, which constrains our ability to comment on transfer of skills beyond the simulated environment. Exam-level features, such as case difficulty and the reduced volume of assessments conducted in particular months (December), may also have influenced performance patterns and partly account for apparent fluctuations or plateaus in the trajectories.

Third, the standardized exam setting itself, where students were required to complete their notes within a fixed time immediately after the encounter, and the stress associated with high-stakes assessment, may have affected how learners prioritized information and managed their time under pressure. Additionally, the cohort's clinical exposure and supervision were shaped by pandemic era conditions, which may differ from those experienced by future cohorts and limit temporal generalizability.

## 5 Conclusion

Our findings endorse the understanding of the complex dynamics of clinical skill development in medical documentation skills, which can also affect other competencies. The analysis reveals areas for improvement and strengths in student performance, with implications for targeted educational interventions to optimize assessment and produce better patient-centered caregivers. The use of simulation-based techniques and technology reconfirmed their role for experiential learning, deliberate practice, and the longitudinal capture of assessment data. Accounting for the impacts of content and context specificity is key to delineating the evolution of students' clinical reasoning skills in the clinical workplace and should be reflected in these specificities being incorporated into the feedback structure, using evidence-based tools such as self-explanation or making thinking visible. Future research studies are essential that include larger diverse cohorts across different institutions to establish the robustness and generalizability of the observed trajectories and clerkship-specific effects. Quasi experimental designs could test the impact of targeted educational interventions—such as explicit teaching on documentation of reasoning, or integrated workplace-based documentation coaching using artificial intelligence (AI)-powered virtual patient simulators—on students' CDS performance in both simulation and clinical settings (35).

## Data Availability

The original contributions presented in the study are included in the article/supplementary material, further inquiries can be directed to the corresponding authors.

## References

[1] Barteelme K Brown M. Development and Evaluation of Students' Skills Critiquing Clinical Documentation. Innov Pharm. (2016) 7:422. doi: 10.24926/iip.v7i1.422

[2] Kusnoor AV Balchandani R Pillow MT Sherman S Ismail N. Near-peers effectively teach clinical documentation skills to early medical students. BMC Med Educ. (2022) 22:712. doi: 10.1186/s12909-022-03790-0 36209076 PMC9548193

[3] Cadieux DC Goldszmidt M. It's not just what you know: junior trainees' approach to follow-up and documentation. Med Educ. (2017) 51:812–25. doi: 10.1111/medu.13286 28418205 PMC5518220

[4] Goldszmidt M Dornan T Lingard L. Progressive collaborative refinement on teams: implications for communication practices. Med Educ. (2014) 48:301–14. doi: 10.1111/medu.12376 24528465

[5] Sinsky CA Willard-Grace R Schutzbank AM Sinsky TA Margolius D Bodenheimer T. In search of joy in practice: a report of 23 high-functioning primary care practices. Ann Fam Med. (2013) 11:272–8. doi: 10.1370/afm.1531 23690328 PMC3659145

[6] van Schaik SM Reeves SA Headrick LA. Exemplary learning environments for the health professions: a vision. Acad Med. (2019) 94:975–82. doi: 10.1097/ACM.0000000000002689 30844927

[7] Biagioli FE Elliot DL Palmer RT Graichen CC Rdesinski RE Ashok KK . The electronic health record objective structured clinical examination: assessing student competency in patient interactions while using the electronic health record. Acad Med. (2017) 92:87–91. doi: 10.1097/ACM.0000000000001276 27332870 PMC5177541

[8] Sim JH Abdul Aziz YF Mansor A Vijayananthan A Foong CC Vadivelu J. Students' performance in the different clinical skills assessed in OSCE: what does it reveal? Med Educ Online. (2015) 20:26185. doi: 10.3402/meo.v20.26185 25697602 PMC4334788

[9] Violato C Cullen MJ Englander R Murray KE Hobday PM Borman-Shoap E . Validity Evidence for Assessing Entrustable Professional Activities During Undergraduate Medical Education. Acad Med. (2021) 96:S70–s5. doi: 10.1097/ACM.0000000000004090 34183605

[10] Rowlands S Tariq A Coverdale S Walker S Wood M. A qualitative investigation into clinical documentation: why do clinicians document the way they do? Health Inf Manag. (2022) 51:126–34. doi: 10.1177/1833358320929776 32643428

[11] Zierler-Brown S Brown TR Chen D Blackburn RW. Clinical documentation for patient care: models, concepts, and liability considerations for pharmacists. Am J Health Syst Pharm. (2007) 64:1851–8. doi: 10.2146/ajhp060682 17724368

[12] Hammoud MM Dalymple JL Christner JG Stewart RA Fisher J Margo K . Medical student documentation in electronic health records: a collaborative statement from the Alliance for Clinical Education. Teach Learn Med. (2012) 24:257–66. doi: 10.1080/10401334.2012.692284 22775791

[13] Yudkowsky R Szauter K. Farewell to the Step 2 clinical skills exam: new opportunities, obligations, and next steps. Acad Med. (2021) 96:1250–3. doi: 10.1097/ACM.0000000000004209 34133347

[14] Harden RM. Assess clinical competence-an overview. Med Teach. (1979) 1:289–96. doi: 10.3109/01421597909014338 24483302

[15] EA Troncon ROD Fernando C. Figueiredo, Eduardo Ferriolli, Lio C, et al. A standardized, structured long-case examination of clinical competence of senior medical students. Medical Teacher. (2000) 22:380–5. doi: 10.1080/014215900409483

[16] Khan KZ Ramachandran S Gaunt K Pushkar P. The Objective Structured Clinical Examination (OSCE): AMEE Guide No. 81 Part I: an historical and theoretical perspective Med Teach. (2013) 35:e1437–46. doi: 10.3109/0142159X.2013.818634 23968323

[17] Tolsgaard MG Pusic MV Sebok-Syer SS Gin B Svendsen MB Syer MD . The fundamentals of Artificial Intelligence in medical education research: AMEE Guide No. 156 Med Teach. (2023) 45:565–73. doi: 10.1080/0142159X.2023.2180340 36862064

[18] Robinson RS. Purposive Sampling. In:Maggino F, editor. Encyclopedia of Quality of Life and Well-Being Research. Cham: Springer International Publishing; 2023. p. 5645–7. doi: 10.1007/978-3-031-17299-1_2337

[19] Makoul G. Essential elements of communication in medical encounters: the Kalamazoo consensus statement. Acad Med. (2001) 76:390–3. doi: 10.1097/00001888-200104000-00021 11299158

[20] Eng K Johnston K Cerda I Kadakia K Mosier-Mills A Vanka A. Patient-Centered Documentation Skills Curriculum for Preclerkship Medical Students in an Open Notes Era. MedEdPORTAL. (2024) 20:11392. doi: 10.15766/mep_2374-8265.11392 38533390 PMC10963659

[21] Alvarez EE Reinhart JM. Use of an interactive online teaching module improved students' ability to write a clinically appropriate SOAP note. J Vet Med Educ. (2020) 47:700–8. doi: 10.3138/jvme.0918-107r 32053056

[22] Daniel M Rencic J Durning SJ Holmboe E Santen SA Lang V . Clinical reasoning assessment methods: a scoping review and practical guidance. Acad Med. (2019) 94:902–12. doi: 10.1097/ACM.0000000000002618 30720527

[23] Suresh KP. An overview of randomization techniques: An unbiased assessment of outcome in clinical research. J Hum Reprod Sci. (2011) 4:8–11. doi: 10.4103/0974-1208.82352 21772732 PMC3136079

[24] Schulz KF Altman DG Moher D CONSORT. 2010 statement: updated guidelines for reporting parallel group randomised trials. Bmj. (2010) 340:c332. doi: 10.1136/bmj.c332 20332509 PMC2844940

[25] Gleeson F AMEE. Medical Education Guide No. 9 Assessment of clinical competence using the Objective Structured Long Examination Record (OSLER) Medical Teacher. (1997) 19:7–14. doi: 10.3109/01421599709019339

[26] Baltagi BH. Econometric Analysis of Panel Data: Springer International Publishing (2021). doi: 10.1007/978-3-030-53953-5

[27] Wooldridge JM. Econometric Analysis of Cross Section and Panel Data, second edition: MIT Press (2010).

[28] Pusic MV Boutis K Hatala R Cook DA. Learning curves in health professions education. Acad Med. (2015) 90:1034–42. doi: 10.1097/ACM.0000000000000681 25806621

[29] Valsamis EM Chouari T O'Dowd-Booth C Rogers B Ricketts D. Learning curves in surgery: variables, analysis and applications. Postgrad Med J. (2018) 94:525–30. doi: 10.1136/postgradmedj-2018-135880 30209180

[30] Norman G Norcini J Bordage G. Competency-based education: milestones or millstones? J Grad Med Educ. (2014) 6:1–6. doi: 10.4300/JGME-D-13-00445.1 PMC3963762 24701301

[31] Osta AD Barnes MM Pessagno R Schwartz A Hirshfield LE. Acculturation needs of pediatric international medical graduates: a qualitative study. Teach Learn Med. (2017) 29:143–52. doi: 10.1080/10401334.2016.1251321 28033485

[32] Durning SJ Artino A Boulet J La RJ Van dVC Arze B . The feasibility, reliability, and validity of a post-encounter form for evaluating clinical reasoning. Med Teach. (2012) 34:30–7. doi: 10.3109/0142159X.2011.590557 22250673

[33] Dong T Saguil A Artino AR Gilliland WR Waechter DM Lopreaito J . Relationship between OSCE scores and other typical medical school performance indicators: a 5-year cohort study. Mil Med. (2012) 177:44–6. doi: 10.7205/MILMED-D-12-00237 23029860

[34] Durning SJ Artino AR. Jr., Pangaro LN, van der Vleuten C, Schuwirth L. Perspective: redefining context in the clinical encounter: implications for research and training in medical education. Acad Med. (2010) 85:894–901. doi: 10.1097/ACM.0b013e3181d7427c 20520047

[35] Boscardin CK Abdulnour RE Gin BC. Macy Foundation Innovation Report Part I: Current Landscape of Artificial Intelligence in Medical Education. Acad Med. (2025) 100(9S Suppl 1):S15–s21. doi: 10.1097/ACM.0000000000006107 40456178

